# Detecting chronic kidney disease by electrocardiography

**DOI:** 10.1038/s43856-023-00306-9

**Published:** 2023-05-26

**Authors:** Jeroen P. Kooman

**Affiliations:** Department of Internal Medicine, Division of Nephrology, Maastricht University Medical Centre, Maastricht, The Netherlands

**Keywords:** Kidney diseases, Kidney

## Abstract

Kooman outlines the findings of a *Communications Medicine* article on deep learning-based detection of chronic kidney disease using ECGs. The author interprets the study’s findings in light of its limitations and outlines key considerations in the clinical implementation of such an approach.

## Teaser

Although chronic kidney disease (CKD) can be detected by relatively simple blood and urine tests, it remains underdiagnosed even in patients at high risk for the disease. Deep learning-based approaches are now being developed to detect CKD based on electrocardiogram (ECG) data.

Chronic kidney disease is a long-term condition involving abnormalities of kidney structure or function that can impact patients’ health. The diagnosis requires either an estimated glomerular filtration rate (eGFR)—a measure of kidney function—below 60 ml/min/1.73 m^2^, based on a prediction formula which includes serum creatinine levels, or the detection of markers of kidney damage, including the presence of albumin in urine (albuminuria). According to eGFR, CKD is subdivided into five different stages, with stage 5 indicating kidney failure. CKD is estimated to be the 12th leading cause of death worldwide. The global prevalence is estimated to exceed 800 million cases^1^. Changes in lifestyle factors and early treatment with angiotensin-converting enzyme inhibitors or angiotensin receptor blockers can delay progression of CKD as well as cardiovascular disease^2^. Nevertheless, many patients with CKD remain undetected, even those with risk factors such as hypertension^2^.

In an article now published in *Communications Medicine*, Holmstrom et al. describe a potential screening approach using a convolutional neural network-based deep learning model to detect CKD from ECG waveforms^3^. Deep learning is a type of machine learning-based artificial intelligence inspired by the human brain, whereby multiple layers of algorithms called convolutional neural networks learn from unstructured data to perform classification or prediction tasks.

The model developed by Holmstrom et al., and similar recently developed approaches^4^, are exciting advances which, if further validated and implemented in the clinic, have the potential to improve detection of patients with CKD using data routinely acquired for other purposes. Here, the findings and clinical implications of the study by Holmstrom et al. are discussed, along with similar studies.

The primary cohort studied by Holmstrom et al. consisted of 111,370 patients and included 247,655 ECGs, of which 100,233 were randomly allocated to the training set and 11,137 to the test set. The model achieved an accuracy of 0.767 in the primary cohort and 0.709 in an external validation cohort. Accuracy was higher with more advanced stages of CKD and in younger patients. This might be explained by the fact that on one hand, ECG alterations are more likely to be prevalent in patients with more advanced CKD, whereas in younger patients, ECG abnormalities due to causes other than CKD are less prevalent.

The definition of CKD was based on ICD-9/10 codes, identifying 7816 patients with a diagnosis of CKD in the primary cohort who had an ECG taken within a 1-year window of a CKD diagnosis. Aside from this relatively long time window, which is of potential relevance because renal function may significantly change over this time period, an important limitation of the study is that data on eGFR were available in only 49% of patients at any point in time. Thus, in 51% of cases, diagnosis only appeared to be dependent on the ICD-9 definition and no connection with the level of kidney function impairment could be made. Moreover, it cannot be excluded that in the population without an ICD-9 diagnosis of CKD, renal impairment might still be prevalent given the fact that CKD is underdiagnosed in the general population. The cohort might also not be representative of the general population, given the relatively high prevalence of end stage kidney failure (2.6%) and because the indication to perform an ECG by itself will introduce selection bias, as the prevalence of CKD is higher in patients with cardiovascular disease. Still, despite these limitations, the fact that model performance was similar in the population in which data on eGFR or albuminuria were present, the consistency of the model performance in different subgroups and comparable performance in the external validation cohort provides confidence that the reported data are sufficiently robust.

Subclinical cardiac abnormalities, such as myocardial fibrosis and left ventricular hypertrophy, are frequently present in patients with kidney dysfunction, even if it is mild^5^. In a population-based cohort, reduced eGFR and albuminuria were associated with an increase in the cardiac biomarkers troponin T and I indicating cardiac damage^6^. In the same cohort, changes in these cardiac biomarkers were associated with ECG alterations suggestive of cardiac damage^7^. In more advanced stages of CKD, ECG abnormalities were also related to cardiovascular mortality^8^. The ability of deep learning models to detect CKD based on ECG changes is therefore likely explained by the high prevalence of these subclinical abnormalities, which result in subtle changes that can remain undetected on routine ECG interpretation. In the study by Holmstrom et al., ECG changes identified to be associated with CKD were primarily observed in the QRS complex and PR interval.

An important possible confounder in the interpretation of ECG changes in patients with CKD are abnormalities in serum potassium. Deep learning models based on ECG interpretation have been validated for the detection of hyperkalemia^9^, and can be predictive of other electrolyte imbalances^10^. While detailed data on electrolytes were not available in the study of Holmstrom et al., the accuracy of the model was similar in patients with and without hyperkalemia in the subgroup where potassium levels were available, which suggest that variations in potassium levels did not have a major influence on the detection of CKD by the model.

In support of the present study, another study by Kwon et al. also assessed the prediction of CKD by ECG analysis using a deep learning model based on convolutional neural networks^4^. In that study, the model was trained to detect an eGFR level of <45 ml/min/1.73 m^2^ (i.e. stage 3B or higher). Accuracy of the model was higher in the study of Kwon et al., which might be due to the fact that data on eGFR were available for all patients or that only patients with a more pronounced decline in renal function were included, with demographic features also included in the model. This study did not, however, evaluate the detection of earlier stage CKD, such as stage 3A disease that is less severe but nevertheless still associated with increased cardiovascular mortality.

Machine learning models based on ECG analysis have also been used in the detection of various types of heart failure, as well as other chronic diseases such as diabetes mellitus^11^. As in CKD, left ventricular hypertrophy and myocardial fibrosis are also common in patients with diabetes and might induce comparable ECG changes. As detailed data on (pre)diabetic status and blood pressure control were missing in the study by Holmstrom et al., the question remains to which extent the model in the study is specific for CKD. As the relation between ECG changes and CKD is likely mediated by subclinical cardiac injury, it may also miss patients with CKD without cardiac damage. However, although this needs to be addressed in future studies, the model may indicate those patients with CKD who are at higher risk for adverse cardiovascular outcomes. Interestingly, in a study where a deep learning model was applied to predict hypo- or hyperkalemia from ECGs, the risk of mortality was higher in those patients where the model indicated abnormalities in serum potassium as compared to laboratory values^12^. This is also relevant for patients with CKD, given the increased prevalence of hyperkalemia and the associated mortality risk in these patients.

Both in the study of Holmstrom et al. as in the study by Kwon et al.^4^, analysis of single lead (I)-ECG resulted in a comparable model performance as compared to the 12-lead ECG. Twelve-lead ECG provide a far more complete of the heart’s electrical activity compared to a single-lead ECG, but is not available in a wearable format. Their comparable performance in this study is of relevance since some newer generation smartwatches are able to perform single lead (I) ECG measurements^13^, which suggests it might one day be possible to detect CKD with a smartwatch in the general population.

At present, given the relatively low sensitivity and specificity, detection of CKD by ECG features alone might not be suitable for widespread implementation as a screening method. However, predictive power may be improved by addition of data from other sources. Recently, it was shown that a machine learning model based on physiological measurements from wearable devices was able to predict to some extent laboratory parameters including blood urea nitrogen^14^. Artificial intelligence models that process more granular data from different sources, including ECGs, might be useful as prescreening tools^15^. These could be followed by more detailed measurements of health status, including blood pressure, and measurements of glucose, renal function and albuminuria.

The remaining question is how, with further validation, the models developed by Holmstrom et al. and Kwon et al. could find their way into future clinical practice. A relatively straightforward way would be to incorporate the model into automated analysis of 12-lead ECG, indicating the need for further analysis in a patient identified at risk of CKD. As ECGs are generally performed for a specific reason, patients have already entered the healthcare system and adding another analysis step might not be too difficult, providing patients have provided consent for this. A different scenario is the one described above, in which data from wearable devices, which may include single lead ECG measurements, are used to identify persons at risk in a general population. In this case, the number of false positive cases is likely high and may be associated with health anxiety and a larger demand on the healthcare system. Moreover, there may be concerns with digital and societal equity as smart wearables may have substantial costs and therefore not be accessible for all socioeconomic groups. For smartwatch-based detection of atrial fibrillation, various ethical and data privacy concerns have also been raised, such as underrepresentation of minority groups in the training data and storage of medical data by commercial parties^16^.

In conclusion, the application of deep learning to detect CKD based on ECG data, as proposed by Holmstrom et al., is an interesting application of a widely used technology will need to be followed by additional validation studies, which should consider integration of different data types. These studies should be performed both in high-risk patients as well as in population-based cohorts, and in patients of different sexes or genders, ethnicities and from different geographical locations. As CKD, even in high-risk populations such as patients with hypertension, remains underdiagnosed, new methods such as the one described in *Communications Medicine* may be a welcome aid by prompting the subsequent use of conventional screening tools. However, even with successful validation, applying these tools on a population basis using wearable sensors would introduce additional ethical, societal, and regulatory challenges that would need to be carefully considered.
